# School Attendance Problems Among Children with Neurodevelopmental Conditions One year Following the Start of the COVID-19 Pandemic

**DOI:** 10.1007/s10803-023-06025-3

**Published:** 2023-07-22

**Authors:** Vasiliki Totsika, Athanasia Kouroupa, Amanda Timmerman, Amanda Allard, Kylie M. Gray, Richard P. Hastings, David Heyne, Glenn A. Melvin, Bruce Tonge

**Affiliations:** 1 Division of Psychiatry, University College London, 149 Tottenham Court Road, Maple House 6th Floor, London, W1T 7NF UK; 2 Centre for Educational Development, Appraisal, and Research (CEDAR), University of Warwick, Coventry, UK; 3 Tavistock & Portman NHS Foundation Trust, London, UK; 4 Council for Disabled Children, National Children’s Bureau, London, UK; 5 Centre for Developmental Psychiatry and Psychology, Department of Psychiatry, Monash University, Melbourne, Australia; 6 Developmental and Educational Psychology Unit, Institute of Psychology, Faculty of Social and Behavioural Sciences, Leiden University, Leiden, The Netherlands; 7 School of Psychology, Faculty of Health, Deakin University, Melbourne, Australia

**Keywords:** School absence, Persistent absence, Refusal, Exclusion, Intellectual disability, Autism

## Abstract

**Purpose:** The present study investigated school absence among 1,076 5–15 year-old children with neurodevelopmental conditions (intellectual disability and/or autism) approximately one year following the start of COVID-19 in the UK. **Methods:** Parents completed an online survey indicating whether their child was absent from school during May 2021 and the reason for each absence. Multi-variable regression models investigated child, family and school variables associated with absenteeism and types of absenteeism. Qualitative data were collected on barriers and facilitators of school attendance. **Results:** During May 2021, 32% of children presented with persistent absence (missing ≥ 10% of school). School refusal and absence due to ill-health were the most frequent types of absence, accounting for 37% and 22% of days missed, respectively. COVID-19 related absence accounted for just 11% of days missed. Child anxiety was associated with overall absenteeism and with days missed because of school refusal. Parent pandemic anxiety and child conduct problems were not associated with school absenteeism. Hyperactivity was associated with lower levels of absenteeism and school refusal but higher levels of school exclusion. A positive parent-teacher relationship was associated with lower levels of absenteeism, school refusal and exclusion. Child unmet need in school was the most frequently reported barrier to attendance while COVID-19 was one of the least frequently reported barriers. **Conclusion:** COVID-19 had a limited impact on school attendance problems during this period. Findings highlighted the role of child mental health in different types of absence and the likely protective role of a positive parent-teacher relationship.

**Supplementary Information:**

The online version contains supplementary material available at 10.1007/s10803-023-06025-3.

The coronavirus pandemic brought many disruptions to children’s education, including the education of children with neurodevelopmental conditions. When schools were open, attendance levels fell compared to pre-pandemic years. Poor school attendance and persistent absence (missing 10% or more of available sessions) have been linked to adverse outcomes for academic progress, mental health, and life chances (Epstein, et al., 2020; Hancock, et al., 2013; Schoeneberger, 2012). Children with neurodevelopmental conditions, such as intellectual disability and/or autism, are less likely to achieve good academic outcomes at school and poorer school attendance increases this risk (Fleming, et al., 2017).

During the first wave of the pandemic, studies indicated a deterioration in child mental health, with notable increases in anxiety and behaviour problems in children with neurodevelopmental conditions (Fridell, Norrman, Girke & Bölte, 2022; Kawaoka, et al., 2021; Samji, et al., 2021; Sideropoulos, et al., 2021; Su, et al., 2021). Changes in mental health were mostly attributed to school disruptions (Genova, Arora, & Botticello, 2021; Kawaoka, et al., 2021). There was some, albeit limited, empirical evidence that returning to school would be a stressor for children with neurodevelopmental conditions, in particular autistic children (Genova, et al., 2021). Parents were also concerned about infection and so happy for their child to stay at home during school closures (Embregts, et al., 2021; Paulauskaite, et al., 2021), or they were less likely to consider academic success important when parents themselves experienced high levels of pandemic anxiety (Cheng, Yang, & Deng, 2021).

Education statistics (UK) reported lower attendance levels for children with special educational needs during 2020-21 compared to other children (DfE, 2022), but the types of absence and the profile of the students most likely to miss school are not known. The assumption that COVID-19 infection or COVID-19 related disruption may be driving higher absence levels requires empirical testing especially since in the USA some data suggested lower COVID-19 transmission and infection in special schools for students with neurodevelopmental conditions compared to other schools in the local area (Sherby, et al., 2021).

Prior to COVID-19, regular school attendance was difficult for some children with neurodevelopmental conditions (Fleming, et al., 2017; John, et al., 2021; Melvin, et al., 2023; Munkhaugen, Gjevik, Pripp, Sphonheim, & Diseth, 2017; Totsika, et al., 2020). Studies to date have either not investigated the association between school absence and child mental health (Totsika, et al., 2020) or found no association (Adams, 2021). In terms of types of absence, school refusal and ill-health were the most frequent types of absence in intellectual disability and autism, respectively (Adams, 2021; Melvin, et al., 2023; Totsika, et al., 2020). School refusal among autistic children has been associated with older age (Adams, 2021; Totsika, et al., 2020), parental unemployment (Adams, 2021), and some aspects of parent and child mental health. An Australian study found that child anxiety was associated only with half-day, rather than full-day, absence (Adams, 2021). Child anxiety does not appear to be associated with absence due to ill-health in autistic children (Adams, 2021), though a recent review called for more research on the association between anxiety and different types of absence (Finning, et al., 2019) as it is likely that child anxiety relates differently to different types of absence.

Before the pandemic, there was mounting evidence of high levels of unmet needs in school, especially for autistic students. Pre- pandemic, parents of autistic children indicated that the main reasons for their children’s school absenteeism were teachers’ lack of autism competence and lack of appropriate adaptations of the school environment including support for learning. Qualitative evidence indicated the mismatch between child needs and school affordances resulted in the escalation of behaviour problems that, in turn, made school exclusion more likely (Brede, Remington, Kenny, Warren & Pellicano, 2017; Sproston, et al., 2017). During the pandemic, the increase in behaviour and mental health problems and the competing time demands (due to COVID-19 risk mitigation measures) resulted in teachers finding supporting students with neurodevelopmental conditions more challenging (Hurwitz, Garman-McClaine & Carlock, 2021; Simo-Pinattella, Goei, Carvalho, & Nelen, 2021). In parallel, some evidence indicated that children with neurodevelopmental conditions, especially those with limited understanding or increased sensory difficulties, found compliance with risk mitigation measures (e.g., mask wearing, COVID-19 testing) difficult (Paulauskaite, et al., 2021; Rogers, et al., 2021).

The aim of the present study was to investigate school absence among children with intellectual disability and/or autism to increase understanding of the types and correlates of absence approximately one year from the start of the COVID-19 pandemic. In the UK education system, students with an intellectual disability and/or autism constitute the majority of students with a formally recognised special educational need (56.3%; DfE 2019). Guided by the theoretical conceptualisation of school absence as a systemic phenomenon (Melvin, et al., 2019) and existing research findings, we collected quantitative data on a range of child, family, and school factors. To empirically test some of the hypothesised impacts of the pandemic, we examined the association between school attendance problems, child mental health, including anxiety, and parent pandemic anxiety. An exploratory approach was adopted when modelling the hypothesised associations with child, family and school factors; while we hypothesised there would be an association between child and parent mental health with school attendance, due to the lack of empirical evidence no specific hypotheses could be made about specific types of school absence. To better understand barriers and facilitators of school attendance during this period, we also collected parent-reported qualitative data.

## Methods

### Study Design

A cross-sectional survey was conducted online with participants recruited across the four UK countries. Study inclusion criteria related to the presence of intellectual disability and/or autism as the child’s main neurodevelopmental condition (as reported by the parent) and a UK residence. Children with co-presenting additional neurodevelopmental conditions and/or physical health problems were eligible for inclusion. Participants could be any adult (18 or over) identifying as a parent or carer of a child aged between 5 and 15 years – a period of compulsory education in the UK [from Reception/Year1 (England/Wales), Year 1 (Northern Ireland) or P1 (Scotland) to Year 10 (England/ Wales), Year 11 (Northern Ireland) or S4 (Scotland)]. The survey was live between June 1st and August 31st 2021 to maximise parents’ ability to remember school absence in May 2021. Participants were recruited with support from several UK-based third-sector organisations (charities) that support families of children with neurodevelopmental conditions. These third-sector organisations advertised the study by distributing a brief leaflet/poster through their social media accounts, mailing lists and newsletters. Interested participants followed a weblink found in the study poster that directed them to the study website; this included detailed study information, a consent form, and a secure link to enter the online survey environment (hosted by Qualtrics©). Survey participation was voluntary and anonymous. Ethical permission was granted by UCL Research Ethics Committee (Project Reference 20633/001). A panel of five expert parent advisors guided the study process, reviewed the survey materials, and contributed to data analysis and data interpretation.

### Participants

Data were collected for 1,076 children with intellectual disability and/or autism. The majority of children were autistic (n = 870, 81%) while 412 children identified as having an intellectual disability (38%). Among participants, 284 children (n = 26%) were autistic and had an intellectual disability whereas 128 children (12%) had an intellectual disability but were not autistic. Children presented with several additional neurodevelopmental conditions (see Table 1). The age of children ranged from 5 to 15 years (M = 10.48 years, SD = 3.00). Most children were boys (n = 718, 67%). Almost one third (n = 280, 26%) reported additional physical health problems including asthma, obesity, epilepsy, dysphagia/eating disorder, heart condition, diabetes, cancer, cystic fibrosis, and Crohn’s disease. Approximately 10% of children (n = 103) were identified as clinically extremely vulnerable by their doctor and 8% (n = 80) had been advised (by health authorities) to shield at any point during the coronavirus pandemic. Clinically extremely vulnerable (CEV) was a UK government classification (Department for Health and Social Care, 2020) used during the pandemic period to identify individuals more susceptible to negative COVID-19 outcomes, some of whom were asked to shield (stay at home). Over 80% (n = 869, 81%) lived in England followed by Scotland (n = 83, 8%), Wales (n = 48, 4%) and Northern Ireland (n = 36, 3%). In terms of educational settings, 600 children (56%) attended a mainstream school, 342 (32%) received special education (special unit in mainstream school, special day school, or residential) and 19 (2%) were in alternative provision (hospital school, pupil referral unit, online, alternative free academy/school).

**Table 1:** *Sociodemographic Characteristics for 1,076 Children with Neurodevelopmental Conditions and their Families*

Characteristic	*n*	*%*
*Child neurodevelopmental conditions*		
Autism	870	81
Intellectual disability	412	39
Attention Deficit Hyperactivity Disorder	258	24
Motor disorders	94	9
Cerebral palsy	23	2
Specific learning disorders	206	19
Communication disorders	245	23
Foetal alcohol syndrome^1^	14	1
*Child physical health*		
Child sensory impairment	75	7
Child mobility problems	135	13
Additional physical problems present^2^	280	26
Child is Clinically Extremely Vulnerable (CEV) ^3^	103	10
Child advised to shield	88	8
*Child educational setting*		
Child attends mainstream school	600	56
Child is in special education	343	32
Child is in alternative provision	19	2
Child has formal identification of special educational needs in educational setting	772	72
*Child demographic characteristics*		
Child is male	718	67
Child ethnicity white	933	90
Child ethnicity mixed/multiple ethnic group	64	6
Child ethnicity Asian/ Asian British	28	3
Child ethnicity Black/ African/ Caribbean/ Black British	7	< 1
*Family sociodemographic characteristics*		
Parent respondent is female	749	70
Survey respondent is mother	1003	93
Respondent is educated below University degree level	709	66
Respondent is not in employment	631	59
Unemployed household	444	41
Single-parent household	256	25
Subjective poverty	80	7
Parent disability	309	29
Parent is Clinically Extremely Vulnerable	65	6

^1^ These do not add up to 100% because children can have more than one neurodevelopmental condition ^2^ Epilepsy, asthma, eating disorders, obesity, cancer, Crohn’s disease, cystic fibrosis, health condition

^3^ Clinically extremely vulnerable (CEV) is a UK government classification (Department for Health and Social Care, 2020) to identify individuals more susceptible to negative COVID-19 outcomes, some of whom were asked to shield (stay at home)

Parents were on average 43 years-old (SD = 7.22, range 24–73 years). About half were educated below university degree level (n = 417, 53%). Approximately 41% of parents reported having a disability (n = 309), while 6% (n = 65) were told by their doctor that they were clinically extremely vulnerable. Slightly over half reported living in a household where at least one parent was in paid employment (n = 632, 58%), while 7% reported finding it quite or very difficult to get by financially (subjective poverty). Table 1 presents participant demographic information.

### Measures

*School absence.* Parents were provided with a list of all weekdays in May 2021 and asked to select the days their child had been absent from school (possible maximum was 19). Using the School Non-Attendance ChecKlist (SNACK; Heyne, et al., 2019), the parent selected one reason from the list of 16 reasons (with a 17th reason classed as Other; see Table 2 and Appendix S1 in Supplementary Material), for each day missed. The SNACK categorises absence into school refusal, truancy, school withdrawal, school exclusion and absence related to health reasons (ill-health or medical appointments). We added two items about COVID-19 absence (see Table 2 and Appendix S1 for the full SNACK). Free-text descriptions provided under ‘Other Reason’ were coded by two researchers following an existing protocol (cf., Totsika et al., 2020). Where appropriate, the free-text descriptions were assigned to a different SNACK item. The remaining ‘Other’ reasons related mostly to child mental health or the child following a reduced timetable in agreement with the school (Table 2). Coded data were checked by the entire research team, and data related to school exclusion were also coded by three parent advisors for external validation.

**Table 2:** *Reasons for School Absence as Measured by SNACK Items and Types of Absenteeism*

	Students N (%^1^)	Min–Max days	%^2^ days missed
1. Child had appointment	60 (15)	0–8	11
2. Child was sick	64 (16)	0–19	11
3. Child refused	181 (45)	0–19	37
4. Child truanted	4 (1)	0–6	< 1
5. Parent gave child day off	20 (5)	0–6	3
6. Parent kept child at home	8 (2)	0–19	< 1
7. Parent arranged extra holidays	4 (1)	0–2	< 1
8. Family emergency (e.g., funeral, hospital admission)	4 (1)	0–1	< 1
9. Family had other difficulties (e.g., car broke down, family’s medical appointment)	4 (1)	0–5	< 1
10. Religious holiday	2 (< 1)	0–2	< 1
11. School closed	17 (4)	0–19	2
12. School sent child home	16 (4)	0–19	2
13. School asked that child stay home	26 (7)	0–19	5
14. Weather	0 (0)	0	0
15. COVID-19 child/family	31 (8)	0–19	6
16. COVID-19 school	27 (7)	0–19	5
17. Other reason	15 (4)	0–19	3
17a. Mental health	5 (1)	0–19	< 1
17b. Reduced timetable	10 (3)	0–18	2
17c. COVID-19 other	2 (< 1)	0–12	< 1
**Health-related absence** (1,2)	118 (29)	0–19	22
**School refusal (3)**	181 (45)	0–19	39
**Truancy (4)**	4 (1)	0–6	< 1
**School withdrawal (5–10)**	32 (8)	0–19	4
**School exclusion (12,13)**	37 (9)	0–19	7
**COVID-19 related absence (15,16,17c)**	58 (14%)	0–19	11

^**1**^ The percentage of students reporting this type of absence amongst students with 1 + day absent n = 403); ^**2**^ The percentage of days lost because of this reason amongst total days missed

*Child mental health.* The 12-item anxiety scale from the parent version of the Developmental Behaviour Checklist (DBC2-Parent; Gray, et al., 2018) was used in the study. Items are rated on a 3-point rating scale (0 = not true as far as I know/ not applicable to my child, 1 = somewhat true or sometimes true, 2 = often true or very true) with total anxiety scores ranging from 0 to 24. Example items are ‘*My child is tense, anxious or worried*’, ‘*My child cries easily for no reason or over small things*’). In the present study, Cronbach’s alpha (0.80) indicated excellent internal consistency.

The Strengths and Difficulties Questionnaire (SDQ; Goodman, 1997) was used to measure conduct problems and hyperactivity; each of these subscales is measured through five items rated on a 0–2 scale with scores ranging from 0 to 10 with higher values indicating higher levels of conduct problems or hyperactivity. In the present study, internal consistency was 0.71 and 0.72 for conduct problems and hyperactivity, respectively.

*Parent-Teacher Relationship.* The Parent-Teacher Relationship subscale from the Parent-Teacher Involvement Questionnaire (PTIQ; Kohl, et al., 2000) is a 6-item measure of the quality of the parent-teacher relationship (e.g., ‘*I enjoy talking to my child’s teacher’*; ‘*I feel my child’s teacher cares about my child’*). Each item is measured on a 5-point scale and items are summed to provide a total score between 0 and 24, where higher values indicate a more positive parent-teacher relationship. Cronbach’s alpha (0.85) suggested excellent internal consistency for the present data.

*Family functioning.* The General Functioning subscale from the McMaster Family Assessment Device (FAD; Epstein, et al., 1983) includes six positively worded items (GF6+; Boterhoven de Haan, et al., 2015) scored on a 4-point Likert scale (e.g., ‘*In times of crisis we can turn to each other for support*’, ‘*We are able to make decisions about how to solve problems*’), 1: strongly agree to 4: strongly disagree), and a total score is the average (range 1–4), with lower scores indicating more positive family functioning. In the present study, Cronbach’s alpha (0.92) suggested excellent internal consistency.

*Parent pandemic anxiety scale.* The Pandemic Anxiety Scale (PAS; McElroy, et al., 2020) is a 7-item scale developed to measure anxiety related to COVID-19 in adults. Items are rated on 5-point Likert scale (0: strongly disagree to 4: strongly agree). The scale captures anxiety about the disease itself (e.g., catching, transmitting, four items) and consequence anxiety (e.g., current finances, economic impact, three items). We used the pandemic disease anxiety subscale (score range 0–16) with higher scores indicating higher levels of anxiety among parents. In the present study, internal consistency was excellent (Cronbach’s alpha: 0.85).

*Barriers and facilitators of school attendance*. Parents were asked to indicate (up to three) barriers and facilitators of school attendance; 780 parents provided free-text data related to barriers and 754 parents about facilitators.

### Approach to Statistical Analysis

Dependent variables were: (a) the total number of school days missed (0–19 range), (b) persistent absence (missing 10% or more of available sessions), and (c) types of school absence (i.e., COVID-19 related, school refusal, school withdrawal, school exclusion, and health-related). Multi-variable models examined the association of each school absence outcome with factors related to the child (gender, age, presence of intellectual disability, ethnicity, additional physical health conditions, clinically extremely vulnerable (CEV) status, anxiety, hyperactivity, conduct problems), the family (number of children in the household, socioeconomic deprivation, parental concern about COVID-19 infection (pandemic anxiety scale), family functioning, parent clinically extremely vulnerable status (CEV), parent disability) and the school (special school vs. other types of school, parent-teacher relationship). Predictor selection processes and sample size adequacy checks for the exploratory modelling approach adopted here are described in detail in Appendix S2 (Online Supplementary Information). In sum, all 17 predictors were included in the models for total days absent and persistent absence as the sample size was adequate for the level of power/margin of error acceptable. A more conservative approach was adopted for modelling types of school absence by selecting predictors whose bivariate correlation (*r*) with the SNACK outcome was ≥ 0.10. This was to compensate for the fact that these models were fitted on fewer participants (children who missed at least one day of school, N = 403) and the fact that some SNACK outcomes showed overdispersion. Regression models for COVID-19 related absence included additional physical problems and family functioning score, while the model for school exclusion included child ethnicity, SDQ hyperactivity score, family functioning scores and parent-teacher relationship. Seven predictors were included in the school refusal model (child gender, presence of intellectual disability, child age, anxiety scores, hyperactivity scores, socioeconomic deprivation, and parent-teacher relationship), while three predictors were included in the health-related absence model (including additional physical health problems, anxiety scores and conduct scores).

All regression results presented below are from multiply imputed data, estimated with robust standard errors. Exponential coefficients are presented that can be interpreted as adjusted risk ratios (ARRs). Data are cross-sectional, so ARRs are best interpreted as adjusted association coefficients rather than causal risk coefficients. For any given predictor, an ARR above 1 indicates an increase in the likelihood of school attendance problems, while an ARR below 1 indicates a decrease in the likelihood of school attendance problems, controlling for all other factors.

Free-text responses about barriers and facilitators were subjected to qualitative analyses in NVivo®: data were initially coded as barriers and facilitators on the basis of agreed definitions of these terms; following this, a bottom-up approach was followed to identify themes and sub-themes within the data. Three researchers worked together to agree on the final coding scheme. Subsequently, data were coded by two researchers with inter-rater reliability estimated for 10% of the data coded independently (kappa = 0.96).

## Results

The mean number of school days missed in May 2021 was 3.5 (SD = 6.45, range 0–19). Approximately 12% (n = 128) of children missed all possible 19 days, while 37.5% (n = 403) missed 1 + days. 32% of children (n = 341) presented with persistent absence (missing ≥ 10% of available sessions). Absence due to school refusal and health reasons were the most frequent types of absence, accounting for 37% and 22% of days missed respectively. COVID-19 related absence accounted for 11% of days missed. Table 2 presents SNACK data.

*Factors associated with school absence and types of school absence.* Table 3 presents the results for total school days missed and persistent absence. Child anxiety, child clinically extremely vulnerable status, child age, parent disability and family socioeconomic deprivation were associated with more days absent while child hyperactivity, number of children in the household, parent clinically extremely vulnerable status, and a more positive parent-teacher relationship were associated with fewer days absent. Child anxiety, child older age, parent disability, and family socioeconomic deprivation were associated with a higher likelihood of persistent absence, while child hyperactivity, poorer family functioning, and a more positive parent-teacher relationship were associated with a lower likelihood of persistent absence (all ARRs in Table 3). Of note, parent pandemic anxiety levels were not associated with total days absent or persistent absence. Additionally, child gender, the presence of intellectual disability, additional physical health problems, conduct problems, and type of school (special school vs. others) were systematically not associated with either outcome.

**Table 3:** *Adjusted Risk Ratios for Total Number of School Days Missed and Persistent Absence*

	Total number of school days missed^1^	Persistent absence (10% or 2 + days missed) ^1^
	ARR (95% CI)	ARR (95% CI)
Child is male	1.17 (0.89, 1.54)	1.11 (0.82, 1.50)
Child has intellectual disability	0.80 (0.59, 1.09)	1.04 (0.75, 1.46)
Child is from a white ethnic group	0.67 (0.43, 1.06)	0.71 (0.46, 1.09)
Child CEV	**1.81 (1.24, 2.63)**	**1.63 (1.02, 2.62)**
Child has additional physical problems	0.99 (0.75, 1.31)	1.08 (0.78, 1.48)
Child age	**1.06 (1.02, 1.11)**	**1.07 (1.02, 1.13)**
Child anxiety (DBC2)	**1.04 (1.01, 1.06)**	**1.07 (1.03, 1.10)**
Hyperactivity (SDQ)	**0.90 (0.85, 0.96)**	**0.91 (0.84, 0.98)**
Conduct problems (SDQ)	1.04 (0.98, 1.11)	1.04 (0.96, 1.11)
Child goes to special school	0.95 (0.70, 1.29)	0.89 (0.63, 1.26)
No of children in household	**0.87 (0.77, 0.99)**	0.88 (0.78, 1.00)
Parent disability	**1.39 (1.08, 1.81)**	**1.65 (1.23, 2.21)**
Parent CEV	**0.62 (0.40, 0.94)**	0.81 (0.46, 1.43)
Family functioning (GF6+)	0.87 (0.61, 1.25)	**0.64 (0.48, 0.85)**
Socioeconomic deprivation ^2^	**1.19 (1.03, 1.38)**	**1.32 (1.13, 1.55)**
Parent Teacher relationship (PTIQ-SF)	**0.92 (0.90, 0.95)**	**0.93 (0.90, 0.96)**
Pandemic Anxiety scale	1.00 (0.97, 1.04)	1.01 (0.97, 1.06)

^1^ Generalised negative binomial regression (total days) and logistic regression (persistent absence); ^2^ A composite of four binary indicators regarding household unemployment, parent education level, subjective poverty and single-parent household

The associations between types of absence, measured by the SNACK, and child, family, and school characteristics were investigated among children who were absent for at least one day (n = 403). Following the process for predictor selection described in Approach to Analysis, COVID-19 related absence was associated only with child additional physical health problems (ARR: 0.23, 95% CI: 0.10, 0.48, *p* < .001), whereby having additional physical health problems was associated with less COVID-19 related absence. School exclusion was associated with ethnic minority status (ARR: 2.79, 95% CI: 1.10, 7.08, *p* = .034), hyperactivity (ARR: 1.65, 95% CI: 1.26, 2.16, *p* < .001) and parent-teacher relationship (ARR: 0.90, 95% CI: 0.81, 0.99, *p* = .037). Ethnic minority status and higher hyperactivity scores were associated with more absence due to exclusion, whereas a more positive parent-teacher relationship was associated with fewer days absent because of exclusion. Health-related absence was more likely in the presence of additional physical health problems (ARR: 1.65, 95% CI: 1.06, 2.57, *p* = .028). Finally, refusal was more likely with increasing child age (ARR: 1.24, 95% CI: 1.15, 1.33, *p* < .001), anxiety (ARR: 1.07, 95% CI: 1.02, 1.12, *p* < .001), and a higher level of socioeconomic deprivation (ARR: 1.41, 95% CI: 1.12, 1.77, *p* = .003). Conversely, refusal was less likely in the presence of an intellectual disability (ARR: 0.59, 95% CI: 0.39, 0.91, *p* = .016), higher hyperactivity scores (ARR: 0.86, 95% CI: 0.79, 0.94, *p* < .001) and a more positive parent-teacher relationship (ARR: 0.93, 95% CI: 0.90, 0.96, *p* < .001).

*Barriers and facilitators of school attendance during the COVID-19 pandemic.* Table 4 presents the barriers and facilitators for school attendance as reported by parents, including the number of times a barrier or facilitator was reported. Tables S1 and S2 in Online Supplementary Information present the same information in more detail including examples of raw data. Parents’ responses included 2,159 barriers grouped into four themes and 1,753 facilitators grouped into three themes. The most frequently reported barrier was the experience of unmet needs in school (n = 664), with the lack of differentiated educational support as the most prominent sub-theme (n = 371). Child health was the second most frequent barrier (n = 659) with half of the health barriers referring to child anxiety (n = 302). “Change” was the third most frequent barrier (n = 438) and referred mostly to change in routines, though changes because of COVID-19 were only a small part of this theme (n = 71). Facilitators tended to mirror barriers. The most frequently reported facilitator was having helpful and effective routines (n = 774) with structure and predictability across home and school environments. Good school provision was the second most frequent facilitator (n = 705) with understanding and adaptation around the child’s additional needs as the largest sub-theme (n = 452). Good parent- or child-teacher relationships were a frequently mentioned facilitator (n = 253), supporting the findings of the regression models.

**Table 4:** *Barriers and facilitators of school attendance in families of children with neurodevelopmental conditions*

Barriers		Facilitators	
**Unmet needs** (n: 664*)		**Good school provision** **(n = 705)**	
Lack of differentiated provision and support (n: 371) No support for social inclusion; not addressing bullying (n: 150) Sensory difficulties related to school (n: 129) Poor teacher relationship and understanding (n: 14)		Understanding and adaptation around child needs (n: 452) Good parent/child – teacher or child–peer relationships (n: 253)	
**Child health problems** **(n: 659)**		**Child mental and physical well-being** **(n: 304)**	
Anxiety (n: 302) Mental health problems (n: 268) Health related medical appointments (n: 89)		Positive emotional well-being and school support for emotional well-being of the child (n: 161) Child is happy (n: 124) Good physical heath and effective medication intake (n: 19)	
**Changes in daily routine** **(n: 438)**		**Effective/Helpful routines** (n*: 774)	
Change in school environment (n: 157) Disruption of home/family routines (n: 120) Change in transport routine (n: 77) COVID-19 related changes (n: 71) Weather changes (n: 13)		Structure and predictability across all environments (n: 536) Effective transport arrangements (n: 141) Good sleep (n: 43) Consistent COVID-19 procedures at school (n: 18) Nice weather (n: 5)	
**Home routines not working** **(n: 398)**			
Morning routine difficulties (n: 190) Lack of sleep (n: 132) Meal/Eating difficulties (n: 26) School journey (n: 50)			

*n reflects the count of references across participants not the number of participants who reported the barrier or facilitator

## Discussion

### School Absence Levels and Types of School Absence

The present study investigated school absenteeism among children with neurodevelopmental conditions, approximately one year following the start of the COVID-19 pandemic in the UK. During one month in which there were no national COVID-19 lockdowns, 32% of 5–15 year-old children missed 10% or more of available school days. This level of persistent absence is similar to levels reported by studies that collected data before COVID-19 from autistic children in the UK (43%; Totsika, et al., 2020) and children with an intellectual disability in Australia (29%; Melvin, et al., 2023). National data on persistent absence levels were not published in England during the pandemic, although daily attendance statistics from the same month indicated variable attendance among students with special educational needs in England (range 61–92%; DfE, 2022, weeks 18–19, 2021).

Investigation of types of absence revealed that school refusal and absence due to ill-health were the most frequently reported types, accounting for 39% and 22% of days missed, respectively. The preponderance of absence related to school refusal and to ill-health is consistent with pre-pandemic findings in children with intellectual disability and/or autism (Adams, 2021; Melvin, et al., 2023; Totsika, et al., 2020). COVID-19 related absence accounted for a small part of overall absence (11% of days missed). In the qualitative data, COVID-19 was one of the least frequently reported barriers to school attendance (Table 4 and S1). In fact, the notion that COVID-19-related changes were a barrier to attendance only arose 71 times, out of a total 2,159 responses. This, in combination with the low levels of COVID-19 related absence, indicates that school attendance among children with neurodevelopmental conditions was primarily driven by non-COVID-19 factors. Indeed, child unmet needs in school was prominent among parent reports of barriers, which suggests that the lack of adaptations in the school environment for the additional or different learning needs of students with neurodevelopmental conditions remains a significant problem. This aligns with pre-pandemic evidence that unmet needs at school are high, and are a key driver of attendance problems and school exclusion (Anderson, 2020; Brede, et al., 2017; Sproston, et al., 2017). In the present data, two of the most frequently reported factors facilitating attendance was ensuring structure and predictability of routines across home and school followed by enhancing teacher understanding of individual needs and ensuring these needs are accommodated within the school environment.

### Child, Family and School Correlates of School Absence

Child mental health and parent pandemic anxiety were a particular focus of the current study, because it was presumed they had a negative impact on school attendance during the pandemic but this had not been tested empirically (Cheng, et al., 2021; Embregts, et al., 2021; Paulauskaite, et al., 2021). A consistent finding was the absence of a significant association between school absence and parent pandemic anxiety levels. This suggests that even if parents experienced anxiety about the pandemic, their anxiety did not interfere with their child’s attendance at school. In contrast, child anxiety was associated with more days absent and persistent absence, unlike a smaller study of autistic children in Australia prior to the pandemic (Adams, 2021). Child anxiety was also associated with school refusal, similar to the forementioned study in Australia (Adams, 2021), but it was not associated with other types of absence such as school withdrawal. This underscores the emotional nature of school refusal, as a feature that differentiates it from other types of absence (Heyne, et al., 2019). Child anxiety features prominently in the qualitative data as one of the most frequent barriers to attendance while the quantitative data shed light on the type of absence with which anxiety is associated. The fact that associations were found between child anxiety and absenteeism but not between parent anxiety and absenteeism might be explained by the measures used. The measure of the parent’s anxiety was about COVID-19 anxiety (McElroy, et al., 2020), while the measure of the child’s anxiety was an overall anxiety measure (Gray, et al., 2018), not COVID-19 specific. Their scores showed overall low correlation (*r*: 0.130), suggesting little overlap between these different anxiety constructs.

Conduct problems were systematically not associated with any of the variables related to absence (i.e., total absence and each type of absence). This is in line with the only other study to examine the association between externalising problems and absence among autistic students (Munkhaugen, et al., 2019). Hyperactivity was associated with lower overall absence and lower school refusal, but higher absence due to school exclusion. Indeed, hyperactivity has been identified as a risk factor for school exclusion in the general population (Fleming, et al., 2017). We found a lower likelihood of persistent absence in the presence of hyperactivity, parent clinically vulnerable status, and poorer family functioning. Findings might indicate that schools were prioritising the attendance of children whose families faced more challenges or that families were more likely to support school attendance if they experienced difficulties.

A positive parent-teacher relationship was associated with fewer days absent, less persistent absence, and a lower likelihood of school exclusion and school refusal. In the qualitative data a positive relationship between families and teachers or schools was one of the most prominent facilitators of attendance. To the best of our knowledge, parent-teacher relationship is a factor not previously investigated in relation to school attendance in families of children with neurodevelopmental conditions. However, research with typically developing students suggests positive parent-teacher relationships are associated with better academic outcomes for children (Deng, et al., 2018; Fu, et al., 2022). It is possible that parents who experience a more positive interaction with teachers will be more inclined to ensure their child attends school. It is also possible that the children of these parents are less likely to refuse to attend school, or to be excluded from school by teachers. In any case, the findings lend support to viewing school absenteeism as a systemic phenomenon (Melvin, et al., 2019). The findings also highlight potential avenues for intervention. Drawing on this study’s qualitative findings, families identified ‘good school provision’ as one of the most significant facilitators of school attendance. Good school provision included adaptation of educational provision around the child’s needs, teacher understanding of neurodevelopmental conditions, and a positive relationship between the family and the school which is underscored by kindness and trust (Table 4 and S2).

### Limitations

Findings need to be interpreted in light of the study’s limitations. Recruitment relied on convenience sampling, thereby limiting the generalisability of study findings. The survey was long and about a third of participants did not complete the final part of the survey. We found that these participants were more likely to experience lower socioeconomic deprivation. While our analyses accounted for this, and results between complete case and multiple imputed models were largely similar, replication studies are needed to determine if the pattern of results reflects the experience of the present sample only. The study was cross-sectional, and no causal relationships should be inferred. Finally, the absence of a comparison group of students without neurodevelopmental conditions substantially limits any conclusions about the specificity of the phenomena under study to children with neurodevelopmental conditions.

## Conclusion

The present study identified that COVID-19 had a limited role in school attendance problems among children with neurodevelopmental conditions, one year following the start of the COVID-19 pandemic in the UK. As we enter the post-pandemic recovery era, findings from the present study would indicate a need to focus on child mental health, in particular anxiety, and perceived level of unmet need as potentially substantial barriers to regular school attendance. At the same time, findings highlight the potentially protective role of parent-teacher relationships. That is, fostering strong and positive parent-teacher relationships will likely benefit school attendance overall and help reduce school refusal and school exclusion, two types of attendance problems with significant implications for the child and the family. Reducing school attendance difficulties in children with a neurodevelopmental condition should remain a priority. Difficulties with school attendance may have been exacerbated during the COVID-19 pandemic, but the assumption that COVID-19 underpinned higher absence levels in children with neurodevelopmental conditions does not withstand the evidence from this study.

### Electronic Supplementary Material

Below is the link to the electronic supplementary material.

Supplementary Material 1

## References

[CR1] Adams, D. (2021). Child and parental mental health as correlates of school non-attendance and school refusal in children on the autism spectrum. *Journal of Autism and Developmental Disorders*, 1–13. 10.1007/s10803-021-05211-5. 10.1007/s10803-021-05211-5 PMC8323746 34331173

[CR2] Anderson, L. (2020). Schooling for pupils with Autism Spectrum Disorder: Parents’ perspectives. *Journal of Autism and Developmental Disorders*, *50*, 4356–4366. 10.1007/s10803-020-04496-2. 32277389 10.1007/s10803-020-04496-2 PMC7677146

[CR3] Brede, J., Remington, A., Kenny, L., Warren, K., & Pellicano, E. (2017). Excluded from school: Autistic students’ experiences of school exclusion and subsequent re-integration into school. *Autism and Developmental Language Impairments*, *2*, 1–20. 10.1177/2396941517737511

[CR4] Cheng, S., Yang, Y., & Deng, M. (2021). Psychological stress and perceived school success among parents of children with developmental disabilities during the COVID-19 pandemic. *Journal of Autism and Developmental Disorders*, 1–8. 10.1007/s10803-021-05209-z. 10.1007/s10803-021-05209-z PMC8318045 34322825

[CR5] de Boterhoven, K. L., Hafekost, J., Lawrence, D., Sawyer, M. G., & Zubrick, S. R. (2015). Reliability and validity of a short version of the general functioning subscale of the McMaster Family Assessment device. *Family process*, *54*(1), 116–123. 10.1111/famp.12113. 25385473 10.1111/famp.12113

[CR6] Deng, L., Zhou, N., Nie, R., Jin, P., Yang, M., & Fang, X. (2018). Parent-teacher partnership and high school students’ development in mainland China: The mediating role of teacher-student relationship. *Asia Pacific Journal of Education*, *38*, 15–31. 10.1080/02188791.2017.1361904. 10.1080/02188791.2017.1361904

[CR7] Department for Education (2022). *Attendance in education and early years settings during the coronavirus (COVID-19) pandemic*. Retrieved March 10, 2022 from: https://explore-education-statistics.service.gov.uk/find-statistics/attendance-in-education-and-early-years-settings-during-the-coronavirus-covid-19-outbreak

[CR8] Department for Education (2021). *Special educational needs and disability: an analysis and summary of data sources* Retrieved March 10, 2022 from: https://www.gov.uk/government/publications/sen-analysis-and-summary-of-data-sources

[CR9] Department for Education (2019). *Special Educational Needs in England: January 2019*. Retrieved November 24, 2020 from: https://assets.publishing.service.gov.uk/government/uploads/system/uploads/attachment_data/file/814244/SEN_2019_Text.docx.pdf

[CR10] Department for Education (2014). *Children with Special Educational Needs 2014: An Analysis*. Retrieved March 10, 2022 from: https://www.gov.uk/government/statistics/children-with-special-educational-needs-an-analysis-2014

[CR11] Department of Health and Social Care (2020). *COVID-19: guidance on protecting people defined on medical grounds as extremely vulnerable*. Retrieved March 10, 2022 from: https://www.gov.uk/government/publications/guidance-on-shielding-and-protecting-extremely-vulnerable-persons-from-covid-19

[CR12] Embregts, P., Heerkens, L., Frielink, N., Giesbers, S., Vromans, L., & Jahoda, A. (2021). Experiences of mothers caring for a child with an intellectual disability during the COVID-19 pandemic in the Netherlands. *Journal of Intellectual Disability Research*, *65*(8), 760–771. 10.1111/jir.12859. 34076326 10.1111/jir.12859 PMC8242374

[CR13] Epstein, N. B., Baldwin, L. M., & Bishop, D. S. (1983). The McMaster family assessment device. *Journal of Marital and Family Therapy*, *9*(2), 171–180. 10.1111/j.1752-0606.1983.tb01497.x. 10.1111/j.1752-0606.1983.tb01497.x

[CR14] Epstein, S., Roberts, E., Sedwick, R., Polling, C., Finning, K., Ford, T., Dutta, R., & Downs, J. (2020). School absenteeism as a risk factor for self-harm and suicidal ideation in children and adolescents: A systematic review and meta-analysis. *European Child and Adolescent Psychiatry*. 10.1007/s00787-019-01327-3. 30989389 10.1007/s00787-019-01327-3 PMC7116080

[CR15] Finning, K., Ukoumunne, O. C., Ford, T., Danielson-Waters, E., Shaw, L., De Jager, R., Stentiford, I., L., & Moore, D. A. (2019). Review: The association between anxiety and poor attendance at school - a systematic review. *Child and Adolescent Mental Health*, *24*(3), 205–216. 10.1111/camh.12322. 32677217 10.1111/camh.12322

[CR16] Fleming, M., Fitton, C. A., Steiner, M., McLay, J. S., Clark, D., King, A., Mackay, D. F., & Pell, J. P. (2017). Educational and health outcomes of children treated for Attention-Deficit/Hyperactivity disorder. *JAMA pediatrics*, *171*, e170691. 10.1001/jamapediatrics.2017.0691. 28459927 10.1001/jamapediatrics.2017.0691 PMC6583483

[CR17] Fridell, A., Norrman, H. N., Girke, L., & Bölte, S. (2022). Effects of the early phase of COVID-19 on the autistic community in Sweden: A qualitative multi-informant study linking to ICF. *International Journal of Environmental Research and Public Health*, *19*(3), 1268. 10.3390/ijerph19031268. 35162290 10.3390/ijerph19031268 PMC8835079

[CR18] Fu, W., Pan, Q., Yuan, Y., & Chen, G. (2022). Longitudinal impact of parent-teacher relationship on middle school students’ academic achievements in China. *Frontiers in Psychology*, *13*, 872301. 10.3389/fpsyg.2022.872301. 36081716 10.3389/fpsyg.2022.872301 PMC9446238

[CR19] Genova, H. M., Arora, A., & Botticello, A. L. (2021). Effects of school closures resulting from COVID-19 in autistic and neurotypical children. *Frontiers in Education*. 10.3389/feduc.2021.761485. 10.3389/feduc.2021.761485

[CR21] Goodman, R. (1997). The Strengths and Difficulties Questionnaire: A Research note. *Journal of Child Psychology and Psychiatry and Allied Disciplines*, *38*, 581–586. 10.1111/j.1469-7610.1997.tb01545.x. 9255702 10.1111/j.1469-7610.1997.tb01545.x

[CR22] Gray, K., Tonge, B., Einfeld, S., Gruber, C., & Klein, A. (2018). *Developmental Behaviour Checklist 2 (DBC2) manual*. Torrance, CA: Western Psychological Services.

[CR23] Hancock, K. J., Shepherd, C. C., Lawrence, D., & Zubrick, S. R. (2013). Student attendance and educational outcomes: Every day counts. *Report for the Department of Education, Employment and Workplace Relations, Canberra*.

[CR24] Heyne, D., Gren Landell, M., Melvin, G., & Gentle-Genitty, C. (2019). Differentiation between school attendance problems: Why and how? *Cognitive and Behavioral Practice*, *26*, 8–34. 10.1016/j.cbpra.2018.03.006

[CR26] Hurwitz, S., Garman-McClaine, B., & Carlock, K. (2021). Special education for students with autism during the COVID-19 pandemic: “Each day brings new challenges. *Autism*, *13623613211035935*, 10.1177/13623613211035935. 10.1177/13623613211035935 34344221

[CR27] John, A., Friedmann, Y., DelPozo-Banos, M., Frizzati, A., Ford, T., & Thapar, A. (2021). Association of school absence and exclusion with recorded neurodevelopmental disorders, mental disorders, or self-harm: A nationwide, retrospective, electronic cohort study of children and young people in Wales, UK. *Lancet Psychiatry*, *9*, 23–34. 10.1016/S2215-0366(21)00367-9. 34826393 10.1016/S2215-0366(21)00367-9 PMC8674147

[CR28] Kawaoka, N., Ohashi, K., Fukuhara, S., Miyachi, T., Asai, T., Imaeda, M., & Saitoh, S. (2021). Impact of school closures due to COVID-19 on children with neurodevelopmental disorders in Japan. *Journal of Autism and Developmental Disorders*, 1–7. 10.1007/s10803-021-05119-0. 10.1007/s10803-021-05119-0 PMC8172360 34081298

[CR29] Kohl, G. O., Lengua, L. J., McMahon, R. J., & Conduct Problems Prevention Research Group. (2000). Parent involvement in school conceptualizing multiple dimensions and their relations with family and demographic risk factors. *Journal of School Psychology*, *38*(6), 501–523. 10.1016/S0022-4405(00)00050-9. 20357900 10.1016/S0022-4405(00)00050-9 PMC2847291

[CR30] McElroy, E., Patalay, P., Moltrecht, B., Shevlin, M., Shum, A., Creswell, C., & Waite, P. (2020). Demographic and health factors associated with pandemic anxiety in the context of COVID-19. *British Journal of Health Psychology*, *25*, 934–944. 10.1111/bjhp.12470. 32860334 10.1111/bjhp.12470

[CR32] Melvin, G., Heyne, D., Gray, K. M., Hastings, R., Totsika, V., Tonge, B., et al. (2019). The kids and teens at School (KiTeS) framework: An Inclusive Nested Framework for understanding School Absenteeism and School attendance problems. *Frontiers in Education*. 10.3389/feduc.2019.00061. 4. 10.3389/feduc.2019.00061

[CR31] Melvin, G. A., Freeman, M., Ashford, L. J., Hastings, R. P., Heyne, D., Tonge, B. J., Bailey, T., Totsika, V., & Gray, K. M., (in press). Types and correlates of school absenteeism among students with intellectual disability.*Journal of intellectual disability research*. 10.1111/jir.13011 36744441

[CR33] Munkhaugen, E. K., Gjevik, E., Pripp, A. H., Sponheim, E., & Diseth, T. H. (2017). School refusal behavior: Are children and adolescents with autism spectrum disorders at a higher risk? *Research in Autism Spectrum Disorders*, 41–42, 31 – 28.

[CR34] Munkhaugen, E. K., Torske, T., Gjevik, E., Nӕrland, T., Pripp, A. H., & Diseth, T. H. (2019). Individual characteristics of students with autism spectrum disorder and school refusal behavior. *Autism*, *23*, 413–423. 29241346 10.1177/1362361317748619

[CR35] Paulauskaite, L., Farris, O., Spencer, H., EPICC-ID group, & Hassiotis, A. (2021). My Son can’t socially Distance or wear a Mask”: How families of preschool children with severe developmental delays and challenging behavior experienced the COVID-19 pandemic. *Journal of Mental Health Research in Intellectual Disabilities*, *14*(2), 225–236. 10.1080/19315864.2021.1874578

[CR36] Rogers, G., Perez-Olivas, G., Stenfert Kroese, B., Patel, V., Murphy, G., Rose, J., Cooper, V., Langdon, P. E., Hiles, S., Clifford, C., & Willner, P. (2021). The experiences of mothers of children and young people with intellectual disabilities during the first COVID-19 lockdown period. *Journal of Applied Research in Intellectual Disabilities*, *34*(6), 1421–1430. 10.1111/jar.12884. 33759291 10.1111/jar.12884 PMC8250127

[CR37] Samji, H., Wu, J., Ladak, A., Vossen, C., Stewart, E., Dove, N., Lond, D., & Snell, G. (2021). Review: Mental health impacts of the COVID-19 pandemic on children and youth - a systematic review. *Child and Adolescent Mental Health*. 10.1111/camh.12501. 34455683 10.1111/camh.12501 PMC8653204

[CR38] Schoeneberger, J. A. (2012). Longitudinal attendance patterns: Developing high school dropouts. The Clearing House. *A Journal of Educational Strategies Issues and Ideas*, *85*, 7–14.

[CR39] Sherby, M. R., Walsh, T. J., Lai, A. M., Neidich, J. A., Balls-Berry, J. E., Morris, S. M., Head, R., Prener, C. G., Newland, J. G., Gurnett, C. A., & the COMPASS-T Study Group. (2021). SARS-CoV-2 screening testing in schools for children with intellectual and developmental disabilities. *Journal of Neurodevelopmental Disorders*, *13*, 31. 10.1186/s11689-021-09376-z. 34465306 10.1186/s11689-021-09376-z PMC8407928

[CR40] Sideropoulos, V., Dukes, D., Hanley, M., Palikara, O., Rhodes, S., Riby, D. M., Samson, A. C., & Van Herwegen, J. (2021). The impact of COVID-19 on anxiety and worries for families of individuals with Special Education needs and disabilities in the UK. *Journal of Autism and Developmental Disorders*, 1–14. 10.1007/s10803-021-05168-5. 10.1007/s10803-021-05168-5 PMC8246131 34196890

[CR41] Simo-Pinatella, D., Goei, S. L., Carvalho, M., & Nelen, M. (2021). Special education teachers’ experiences of addressing challenging behaviour during the pandemic. *European Journal of Special Needs Education*. 10.1080/08856257.2021.1963152. 10.1080/08856257.2021.1963152

[CR42] Sproston, K., Sedgewick, F., & Crane, L. (2017). Autistic girls and school exclusion: Perspectives of students and their parents. *Autism and Developmental Language Impairments*, *2*, 1–14. 10.1177/2396941517706172

[CR43] Su, X. Y., Cai, R. Y., Uljarevic, M., Van Herwegen, J., Dukes, D., Yang, Y., Peng, X., & Samson, A. C. (2021). Brief report: A cross-sectional study of anxiety levels and concerns of chinese families of children with special Educational needs and disabilities post-first-wave of COVID-19. *Frontiers in Psychiatry*, *12*, 10.3389/fpsyt.2021.708465. 10.3389/fpsyt.2021.708465 PMC8488298 34616315

[CR44] Totsika, V., Hastings, R. P., Dutton, Y., Worsley, A., Melvin, G., Gray, K., Tonge, B., & Heyne, D. (2020). Types and correlates of school non-attendance in students with autism spectrum disorders. *Autism*, *24*, 1639–1649. 10.1177/1362361320916967. 32419486 10.1177/1362361320916967 PMC7545649

