# Supplementary material for: School Attendance Problems Among Children with Neurodevelopmental Conditions One year Following the Start of the COVID-19 Pandemic
Source: J Autism Dev Disord. 2023 Jul 22;54(8):2998–3007. doi: 10.1007/s10803-023-06025-3 (PMC11300561; doi:10.1007/s10803-023-06025-3)
Supplement: Supplementary file 1 — Supplementary Material 1 [file 10803_2023_6025_MOESM1_ESM.docx]

**Supplementary Information for “School attendance problems among children with neurodevelopmental conditions one year following the start of the COVID-19 pandemic”**

**Appendix S1: School Attendance Checklist (SNACK)- COVID-19 (UK adaptation 2021 of Heyne et al., 2019)**

| ***Reason*** | ***Examples*** | ***Tick*** |
| --- | --- | --- |
| ***My child*** |  |  |
| 1. had an appointment | - a doctor’s appointment - an appointment with a specialist |  |
| 1. was sick | - had a cold or flu; had asthma - was in hospital |  |
| 1. was reluctant or refused | - he/she said it was hard to go to school or stay there the whole day - he/she seemed upset/anxious/scared about school  \|  \| \| --- \| |  |
| 1. skipped/wagged/truanted | - he/she headed to school but did not arrive there - he/she left the school without permission |  |
| **I or my partner** |  |  |
| 1. gave my child a day off | - to give him/her a rest |  |
| 1. kept my child home for other reasons | - so he/she could help out at home - because school is not helping him / her |  |
| 1. arranged extra holidays | - to take a family holiday during school time |  |
| **Our family** |  |  |
| 1. had an urgent situation | - a funeral - someone in the family was taken to hospital |  |
| 1. had other difficulties | - the car broke down - someone in the family had a medical appointment |  |
| 1. had a religious holiday or cultural observance | - End of Ramadan - Jewish holidays - Buddha Day/Vesak |  |
| **The school** |  |  |
| 1. was closed | - public holiday/bank holiday; term holidays - curriculum day / teacher training day/ teacher strike - school problem (e.g., flood, failed heating) |  |
| 1. sent my child home due to his/ her behaviour | - he/she was suspended or expelled from school - he/she was asked to leave school for the remainder of the day |  |
| 1. asked that my child stay away from school | - because the school could not take care of my child’s needs - because the school could not keep my child safe at school |  |
| **Other** |  |  |
| 1. weather conditions | - snow, floods - fire |  |
| 1. COVID-19 | - my child was shielding, someone in our family was shielding; - my child was isolating |  |
| 1. COVID-19 - school | - the school was shut due to COVID-19 - the whole class/year was isolating - the school asked that we keep our child at home because of school difficulties with class bubble size, transportation, or staff numbers |  |
| 1. Something else. Please describe in the space provided |  |  |

**Appendix S2: Approach to Analysis**

School non-attendance was defined as: (a) the total number of school days missed (0–19 range) and (b) persistent absence (missing 10% or more of available sessions; DfE, 2019). All analyses were conducted in IBM SPSS 28.0 ®. Multi-variable models examined the association of each-school non-attendance outcome with factors related to the child (gender, age, ID presence, ethnicity, additional physical health conditions, clinically extremely vulnerable (CEV) status, anxiety, hyperactivity, conduct problems), the family (number of children in the household, socioeconomic deprivation, parental concern about covid-19 infection [pandemic anxiety scale], family functioning, parent clinically extremely vulnerable status (CEV), parent disability) and the school (special school vs other types of school, parent-teacher relationship). A logistic regression model was fitted for persistent absence and a negative binomial regression was fitted for total days absent. To determine the adequacy of the sample size for the given number of predictors (17) in this exploratory study, we used the Riley et al. (2020) approach for persistent absence. This approach indicated that a sample size of 753 was required to model this binary outcome with a small margin of error (<0.05). There is no standardised approach to determine the adequacy of the sample size in exploratory multi-variable negative binomial models (c.f., Zhu & Lakkis, 2014), therefore we consulted two alternative approaches. G*power (Faul et al., 2009) provides a priori and post-hoc power estimates for Poisson multi-variable regression, while STATA power_tworates_zhu (Chatfield, 2021) can determine the sample size when comparing two negative binomial rates based on an approach developed by Zhu and Lakkis (2014). While neither scenario is similar to our design, taken together these approaches provide tentative indications of the adequacy of the existing sample size. For 5% alpha and 80% power, the existing sample is adequate to detect moderate to large effects associated with total days absent.

SNACK scores described types of absence: COVID-19 related absence, school refusal, truancy, school withdrawal, school exclusion, and health-related absence. Among children who missed at least one day of school (N=403), we examined the association between each type of absence with child, family and school predictors. Negative binomial regression models were fitted for these count outcomes. To determine the adequacy of the sample size, we considered information from G*power and the Zhu & Lakkis (2014) power analyses as above. The sample size was adequate to detect small effects for all SNACK outcomes modelled in multivariable poisson model, and for detecting moderate to large effects in 2-group negative binomial comparisons. However, where dispersion was greater (exclusion and COVID-19 related absence) only very large between-group differences (i.e, binary predictors) would be detected with power 80%. For this, we adopted a more conservative approach in potential predictor selection, by selecting predictors whose bivariate correlation (*r*) with the SNACK outcome was ≥ 0.10. Regression models for COVID-19 related absence included additional physical problems and family functioning score, while the model for school exclusion included child ethnicity, SDQ hyperactivity score, family functioning scores and parent-teacher relationship. Seven predictors were included in the school refusal model (child gender, ID, child age, anxiety scores, hyperactivity scores, socioeconomic deprivation, and parent-teacher relationship), while three predictors were included in the health-related absence model (additional physical health problems, anxiety scores and conduct scores). Following estimation, post-hoc analyses in G*power indicated all SNACK outcome models had achieved power of 1.

Overall, 7.3% data values were missing across the database, with 29.5% participants having one or more missing data points. The amount of missing data increased as the survey progressed, with smaller amounts of missing data in variables at the first (child characteristics) and second (school absence) parts of the survey but larger amounts of missing data in the final part (variables related to family). Little’s MCAR test indicated that data were not missing completely at random (MCAR): *χ^2^:* 574.23*, p*<.001. An examination of the pattern of missing data in relation to outcomes and predictors indicated low association of missingness with study outcomes and all predictors (all *r* coefficients below 0.4; Schafer & Graham, 2002), except for socioeconomic deprivation (*r: -.*681) which indicated that participants from less deprived families were more likely to not complete the final part of the survey. Missing Not at Random (MNAR) among potential predictor variables could not be ruled out so multiple imputation under a fully conditional specification framework (MI-FCS) was performed (Ward, Axon, & Gebregziabher, 2020). Auxiliary variables selected for their association with variables with high levels of missing data and the likelihood of missing data were included in the database to maximise the plausibility of random missingness (MAR). Ten imputed datasets were created using an iterative Markov chain Monte Carlo to impute missing values in the covariates/predictors while all outcomes were completely observed. A pooled database was used for analysis; regression coefficients from complete-case models and multiply imputed data were largely similar (except for the exclusion models), while confidence intervals tended to be narrower suggesting more precise estimation in the multiple imputation data. All regression results presented in the paper are from models using the multiple imputation data. Regression models were estimated with robust standard errors, and exponential coefficients are presented that can be interpreted as adjusted risk ratios (ARRs).

Finally, parent-reported barriers and facilitators of school attendance were subjected to qualitative analysis in NVivo®: data were initially coded as barriers and facilitators on the basis of agreed definitions of these terms; following this, a bottom-up approach was followed to identify themes and sub-themes within the data. Three researchers worked together to agree on the final coding scheme. Researchers (two-doctoral level and one Master’s level researcher) had received formal training on the coding of qualitative data and the use of NVivo® from independent experts in qualitative analysis prior to the study. For the purposes of the present study, they were supervised by a fourth researcher, experienced in qualitative analyses, who guided the team throughout the process of coding, including initial familiarisation with the data, code refinement and finalising the themes and sub-themes. As with the coding of free-text SNACK data, external validation of emerging and final codes was sought from members of the expert parent advisory panel. Researchers worked in pairs and as a group to ensure consistency in coding and, once the final coding scheme was agreed, data were coded by two researchers with inter-rater reliability estimated for 10% of the data coded independently (kappa= 0.96).

**Table S1**

*Detailed Data on Parent-Reported Barriers for School Attendance*

| **Overarching Theme** | **Subthemes** | **Definition** | **Examples** |
| --- | --- | --- | --- |
| Unmet needs at school  (n*: 664) | Lack of differentiated provision and support (n: 371) | Absence of recognition and/or acknowledgement of the needs of the child by school staff regarding learning expectations, use of unstructured time, implementation of special education needs plan, and/or child’s safety. | “Lack of understanding by teachers”  “Too long in the classroom”  “Unmet needs in unstructured times”  “Learning expectations”  “Lack of support”  “Child’s lack of danger awareness”  “Poor EHCP” |
|  | No support for social inclusion; not addressing bullying (n: 150) | Absence of actions to create and/or enhance social interaction with peers in school and lack of support to address and/or prevent incidents of bullying. | “School not keeping my child safe; constant bullying not dealt with, withholding information on physical attacks on my child”  “Socialising or seeing people and fear of failure”  “Unkind children”  “Fear of being bullied”  “Loneliness”  “Confidence, feeling she's never picked by peers for teams” |
|  | Sensory difficulties related to school (n: 129) | Lack of provision to minimise the impact of overwhelming stimuli (e.g., noise) and sensory discomfort by wearing a uniform in school/class. | “Unsuitable school environment e.g., noise /chaos”  “Sensory difficulties with the school environment”  “Hard finding comfortable clothing school uniform”  “Sensory issues - my son is easily upset by the feeling of certain fabrics, blisters, itches”  “Sensory overload”  “Loud members of the classroom” |
|  | Poor teacher relationship and understanding (n: 14) | Poor teacher-parent communication, poor parent-teacher relationship, poor child-teacher relationship; teachers’ limited understanding of autism and child needs. | “School teachers’ attitude towards autism”  “School not telling us that our clinically extremely vulnerable child has been exposed to risk”  “Lack of trust in school staff”  “Lack of communication from school”  “Poor relationships with teachers”  “Negativity from schoolteachers”  “Barriers created by school being unable to communicate appropriately with autistic parent” |
| Child health problems  (n: 659) | Anxiety (n: 302) | Presentation of anxiety about academic performance, (implied) separation anxiety, social relationships at school, transitions. | “Social anxiety, general anxiety”  “Being away for mum, secure person”  “Anxiety about ability to do work at school”  “Anxiety about being in classroom”  “Anxiety about other children crying” |
|  | Mental health problems (n: 268) | Presence of diagnosed/undiagnosed mental health conditions other than anxiety including emotional and behavioural manifestations and refusal going to school. | “Self-harm”  “Panic attacks”  “Depression”  “My child does not like going to school and does not like her teacher”  “School trauma leading to mental health problems”  “School refusal” |
|  | Health related medical appointments (n: 89) | Disruptions due to the need to attend medical appointments, poor physical health and/or accidents at school or home. | “Medical appointments”  “Seizures”  “Side effects of medications e.g., many epilepsy drugs cause drowsiness”  “Feeling sick”  “Vomiting” |
| Changes in daily routine  (n: 438) | Change in school environment (n: 157) | Unhelpful events taking place in school (e.g., timetable changes, teachers on leave, class change, non-uniform day etc.). | “Change to routine such as non-uniform day”  “Change of teachers”  “Unfamiliar staff greeting her”  “Devices not working”  “Uncertainty/last minute changes and disruption - particularly having new rules issued last minute”  “Something out of the ordinary happening e.g., play rehearsal, concert, end of term party” |
|  | Disruption of home/family routines (n: 120) | Unexpected changes that disrupt the child’s routine at home. | “Different school terms to sibling's mainstream school”  “Change at home e.g., different parent organising child in morning”  “Events at home e.g., visitors staying, siblings not attending for valid reasons (e.g., sixth form free periods)”  “Newborn”  “My son being off on Fridays and taking him to my daughters’ nursery” |
|  | Change in transport routine (n: 77) | Unexpected changes related to the mean of transport from/to school including the driver or adult who accompanies the child. | “His bus being late”  “Bus not arrived”  “Different escort on the bus”  “Parking around the school”  “Change in amount of time driving in the car” |
|  | COVID-19 related changes (n: 71) | Changes in school or at home because of COVID-19 or COVID-19 risk mitigation measures | “Getting a covid test done for a child with a severe disability is really traumatic but the alternative is no school for 10 days”  “Bubble closures”  “School has its own rules on what is a COVID symptom and seem to want children to stay off school for the tiniest reason even when they are well”  “Concerns re Covid - schools are not safe”  “Local covid cases are high”  “Frightened of bringing covid home” |
|  | Weather changes (n: 13) | Changes related to weather that disturb the child. | “Change in weather e.g., trying to dress him in shorts in the summer when he is used to trousers”  “Rain, hates getting wet”  “Threat of thunderstorm”  “Weather - reacts badly to bad weather” |
| Home routines not working  (n: 398) | Morning routine (n: 190) | Child difficulties understanding time and managing tasks in the morning. | “Refusal to get dressed + washed / teeth / hair”  “Waking up and getting moving”  “Personal Care - refusing to shower”  “Timekeeping - my son is very distractable and gets very late, he cannot manage his time”  “Slow to get ready”  “Struggles with organising herself” |
|  | Lack of sleep (n: 132) | Disruptive sleep pattern. | “No sleep night before”  “Up too early as doesn't sleep much”  “Getting out of bed early”  “Lack of sleep/sleep apnea”  “Tiredness due to no sleep” |
|  | Meal/Eating (n: 26) | Problems with eating breakfast (e.g., food and/or fluids denial, too long to eat). | “Eating breakfast”  “Hard to get breakfast in him before school as can take ages, will say he is full if you rush him”  “Thought of eating”  “Run out of breakfast cereal”  “Refusing to eat/drink” |
|  | School journey (n: 50) | Lack of child’s adjustment to standard events distance from/to school, problems with accessibility, siblings going to another school. | “Distance to school”  “School inaccessible”  “Leaving home”  “Location 1 hour drive from home”  “Getting out of the door”  “It’s far away and the roads are dangerous”  “Stopping to drop off sibling at nursery” |

**Table S2**

*Detailed Data on* *Parent-Reported Facilitators of School Attendance*.

| **Overarching Theme** | **Subthemes** | **Definition & Examples** |  |
| --- | --- | --- | --- |
| Effective/Helpful routines  (n*: 774) | Structure and predictability across all environments (n: 536) | Consistent and familiar home/school routines followed regularly across settings. | “Set routine”  “Consistent approach - meet & greet”  “Pizza Friday”  “Same entrance”  “Clear rules and expectations”  “Playing a game like rock paper scissors on the way to school” |
|  | Effective transport arrangements (n: 141) | Helpful transport arrangements arranged by the local council, school, and parents. | “Enjoys getting picked up by her dad who brings her scooter”  “Transport provision. we used to have a taxi that was paid for by the council”  “Being able to park right by the school for safety reasons”  “School transport with escort”  “Driven in by own car” |
|  | Good sleep (n: 43) | The child is rested as a result of good sleep or good sleep routines. | “Good sleep night before”  “Sleeps well”  “Well rested”  “Not staying overnight” |
|  | Consistent COVID-19 procedures at school (n: 18) | COVID-19 measures implemented appropriately and consistently in school. | “Being in a class bubble”  “Face masks”  “Adults/pupils following the rules e.g., mask wearing”  “Social distancing”  “Staff vaccination and Lateral Flow testing” |
|  | Nice weather (n: 5) | Nice weather conditions. | “Sunny weather”  “When it's not raining”  “Sunny day can be outside” |
| Good school provision (n*: 705) | Understanding and adaptation around child needs (n: 452) | Understanding and implementation of good practices by school staff (e.g., understanding of individual interests) to accommodate individual needs (additional space, sensory aids, transition items). | “Letting him take his Thomas mini to school”  “When the teacher engages with her about one of her interests”  “New staff being informed of my son’s needs”  “Teaching Assistant waiting with fidget toy and not waiting in line but going straight into class”  “Sensory breaks”  “Being able to attend but not in the classroom”  “More comfortable uniform”  “School working with family and good communication on specific issues (e.g., hates sports day but we prepare him ahead of time e.g., social story)” |
|  | Good parent/child – teacher or child–peer relationships (n: 253) | Positive relationships between school staff and the child or parents; positive social interactions and relationships with friends in school. | “Kindness form teachers”  “Bubble of friends”  “Trusting his key worker”  “Having a suitable school”  “Peer buddy system” |
| Mental and physical well-being of the child  (n: 304) | Positive emotional well-being and school support for emotional well-being of the child (n: 161) | Support or active intervention provided within school to enable the child to feel well within a calm and supportive environment. | “When the pastoral support is right”  “Daily occupational therapy being provided each day upon arrival in school”  “Feeling cared for and supported at school”  “Knowing she can go and see her Head of year or to a quiet space if needed”  “Sibling at same school provides reassurance” |
|  | Child is happy (n: 124) | The child shows preference for certain subjects/days/events/teachers. | “Something positive happening e.g., doughnut stall”  “Having a happy school environment so he can look forward to things”  “Preferred teachers/lesson subjects”  “Fun days in school”  “Being in a good mood” |
|  | Good physical heath and effective medication intake (n: 19) | The child is in good health or health-related problems are managed with medication. | “No illness”  “No appointments in school time”  “Medication”  “No injury from falling due to seizure activity”  “No asthmatic episode” |

*n reflects the count of references across participants not the number of participants who reported a facilitator.

References in Supplementary Information

Chatfield, M. (2019). POWER_TWORATES_ZHU: Stata module to calculate sample size power for a two-sample test of rates. *Statistical Software Components* S458624, Boston College Department of Economics, revised 23 Aug 2021.

Department for Education (2019). *A Guide to Absence Statistics*. London: Department for Education. Retrieved March 10, 2022 from: <https://www.gov.uk/government/publications/absence-statistics-guide>

Faul, F., Erdfelder, E., Buchner, A., & Lang, A.-G. (2009). Statistical power analyses using G * Power 3.1: Tests for correlation and regression analyses. Behavior Research Methods, 41 , 1149-1160.

Heyne, D., Gren Landell, M., Melvin, G., & Gentle-Genitty, C. (2019). Differentiation between school attendance problems: Why and how? *Cognitive and Behavioral Practice*, 26, 8-34.

Riley, R. D., Ensor, J., Snell, K., Harrell, F. E., Jr, Martin, G. P., Reitsma, J. B., Moons, K.G.M., Collins, G., & van Smeden, M. (2020). Calculating the sample size required for developing a clinical prediction model. *BMJ (Clinical research ed.)*, 368, m441. <https://doi.org/10.1136/bmj.m441>

Schafer, J. L., & Graham, J. W. (2002). Missing data: our view of the state of the art. *Psychological Methods*, 7(2), 147–177.

Ward, R. C., Axon, R. N., & Gebregziabher, M. (2020). Approaches for missing covariate data in logistic regression with MNAR sensitivity analyses. *Biometrical Journal,* 62(4), 1025–1037. https://doi.org/10.1002/bimj.201900117

Zhu, H., & Lakkis, H. (2014). Sample size calculation for comparing two negative binomial rates. *Statistics in Medicine*, 33(3), 376–387. https://doi.org/10.1002/sim.5947
